# The Education of Defective Children

**Published:** 1904-09-10

**Authors:** 


### HOSPITAL ADMINISTRATION.

### CONSTRUCTION AND ECONOMICS.

### THE EDUCATION OF DEFECTIVE CHILDREN.

It is not pleasant to look back over the education of the past, and think how much mentally deficient children must have suffered from even well-meaning teachers. The palpably imbecile child might be fortunate enough to enjoy a little wholesome neglect, but harshness, amounting to positive cruelty, was the lot of the boy or girl whose deficiencies were a shade less obvious. With all the fault-finding—some of it justifiable enough—which modern education has had to endure, it must be set down to the credit of our educators that they try to discover the respective capacities and incapacities of the children they have to do with. Apparent inattention or disobedience has often been proved to be deafness, defects of vision have been shown to be the cause of much that passed for carelessness, and what was punished as incorrigible obstinacy and contempt for authority has been found to be due to the limitations of an intelligence below the average. For such children the ordinary teacher can do little, especially when the defective is one of a class of normal children. The regulations recently published by the Board of Education are evidence of the belief of the authorities—a belief which is founded on experience—that a child who, placed in an ordinary class in an elementary school, would be only a hindrance to his companions and would gain little or nothing himself may, if properly taught, attain a fair amount of education. The main point in these regulations are instructions as to the size of the classes and the nature of the teaching. Defective children cannot well be taught in the mass; they require far more individual attention than normal boys and girls. Therefore the number of children in each class is limited to 20 for ordinary teaching and 10 for manual instruction. Manual instruction has a prominent place in the curriculum of the school for defective children, partly, no doubt, because many who cannot reach any great degree of proficiency in intellectual work may do very well in handicraft, but partly also because the more limited mind can grasp the abstract only through the concrete. In the infant-room children are taught the elementary rules of arithmetic, for example, by means of actual objects, and for defective children the methods of the infant-room must be kept up in maturer years.

Doubtless this fact accounts for the preference given to those who have the certificates of the Froabel Society as teachers in schools for defective children. The methods of the kindergarten are those best adapted for these limited minds. As there are between children who may all be classed as defective greater differences than there are between normal children, it is impossible to fix a definite age-limit for any of the classes in the school where they are taught. Nominally they are divided into "younger" and "elder" children, but this does not imply that all in the former division are the juniors of the others. Though advancing years will bring progress, it is likely that there will always be children who are inferior in attainments to others who are younger in years than themselves. This is due partly to natural inferiority of capacity but may also be caused by delay in going to the special school. No child is admitted to the school for defectives under the age of seven, but it may very easily happen that through teachers not perceiving that the child's backwardness is due to real lack of capacity, or to parents refusing to accept and admit the fact, the child may go on struggling drearily and uselessly for a year or two after this. At whatever age the child enters the school, he must leave it when he is 16. All pupils do not stay the full time possible at the special school, for it is found that some, after the first steps have been made easy for them there, are able to return to the ordinary school and do reasonably well among normal children. Examinations are held to test the children's fitness for such a removal, and if parents think that their children have attained a state of mental and physical health which would justify such a change, they may demand an examination of their children, but not oftener than once in six months.

For epileptic children, also, special schools are provided, but these are certified as suitable only if—besides elementary instruction—board, lodging, and medical treatment are provided for the pupils. A more advanced curriculum, both in ordinary and in manual instruction, is permitted for the epileptics than for the defective, and has a threefold object—to train the pupils in hand, eye, and brain, to teach them to earn their living, and to improve their health. The trades to be taught are, however, limited to some extent by the proviso that care is to be taken to avoid the use of dangerous tools and machinery. It is recommended also that schools for epileptics should be situated in the country, and, where it is possible, should be built entirely on one floor, so as to avoid the need for staircases. With pupils liable to sudden fits and loss of consciousness, such precautions are necessary. Another point of importance is the limitation of size. The Board of Education refuses to give its certificate to any institution, at least to any established in the present century, which provides for the reception of more than 30 epileptic children.

If the care given to each is rewarded with success; if the defective children improve in mind and the epileptics in body, there is no reason why individuals of both these classes of afflicted should not become more or less self-supporting and, therefore, happy beings. The boys among them will be taught shoemaking, tailoring, basket-making, chair-caning, matmaking, and—probably best of all—gardening and farm-work. The girls also are taught gardening, in addition to cookery, laundry-work, needlework, and the general art of housewifery. Thus equipped they should find some niche in the world's economy, even though they may never be able to compete on equal terms with their healthier and saner mates.

